# Asynchronous division at 4–8-cell stage of preimplantation embryos affects live birth through ICM/TE differentiation

**DOI:** 10.1038/s41598-022-13646-8

**Published:** 2022-06-07

**Authors:** Daisuke Mashiko, Zenki Ikeda, Mikiko Tokoro, Yu Hatano, Tatsuma Yao, Tetsuya J. Kobayashi, Noritaka Fukunaga, Yoshimasa Asada, Kazuo Yamagata

**Affiliations:** 1 Graduate School of Biology-Oriented Science and Technology, Kindai University, Kinokawa, Wakayama 649-6493 Japan; 2 Asada Institute for Reproductive Medicine, Asada Ladies Clinic, Nagoya, Aichi 450-0002 Japan; 3 Research and Development Center, Fuso Pharmaceutical Industries, Ltd., Joto, Osaka 536-8523 Japan; 4 Institute of Industrial Science, The University of Tokyo, Tokyo, 153-8505 Japan

**Keywords:** Embryogenesis, Embryology

## Abstract

To improve the performance of assisted reproductive technology, it is necessary to find an indicator that can identify and select embryos that will be born or be aborted. We searched for indicators of embryo selection by comparing born/abort mouse embryos. We found that asynchronous embryos during the 4–8-cell stage were predisposed to be aborted. In asynchronous mouse embryos, the nuclear translocation of YAP1 in some blastomeres and compaction were delayed, and the number of ICMs was reduced. Hence, it is possible that asynchronous embryos have abnormal differentiation. When the synchrony of human embryos was observed, it was confirmed that embryos that did not reach clinical pregnancy had asynchrony as in mice. This could make synchrony a universal indicator common to all animal species.

## Introduction

The number of infertile patients in the world is increasing; in 1990, there were 42 million pairs, while in 2010, there were 48.5 million pairs^1,2^. With the increasing number of treatments using assisted reproductive technology, opportunities to culture embryos in vitro are increasing. While multiple embryos are cultured, single embryo transfer is performed to avoid the adverse health effects of multiple pregnancies on the mother and children^3^. Therefore, when transplanting an embryo, it is necessary to select one embryo from multiple cultured embryos.

Gardner criteria, which are defined by the expansion stage of the blastocyst and the appearance of inner cell mass (ICM) and trophectoderm (TE)^4,5^ are used for selecting embryos. The pregnancy rate following the selection and transplantation of a fertilized egg is 20–30%^6^. To achieve a higher pregnancy rate, indicators that can predict the prognosis of embryos in combination with these criteria are required. Indicators can be found by comparing embryos that were born and those that were not born, and the effectiveness of the indicator can be confirmed by transplantation based on the selection of embryos by the indicator. Retrospective analysis of human embryos has revealed some prospective indicators^7–13^, but most studies of human embryos did not investigate why the index could determine the outcome. Therefore, it is not clear what these proposed indices have been evaluating.

We have previously constructed a minimally invasive fluorescent live-cell imaging system for preimplantation embryos, which is applicable to various animal species (mouse:^14^, bovine:^15^. By combining this system with a single embryo transfer (a method in which a single blastocyst is transplanted to a uterus of a pseudo-pregnant female mouse), it is possible to search for effective parameters for blastocyst selection. We previously applied this combination to mouse embryos and found that abnormal chromosome segregation during early division can affect blastocyst development but does not necessarily affect developmental capacity after the blastocyst stage^16^. The purpose of this study was to search for a reliable embryo selection indicator by fluorescent live-imaging of mouse embryos, to verify the indicator by observing movies of human embryos, and to investigate why the index can separate between born/abort outcomes.

In the present study, we recorded videos of mouse embryos microinjected with mRNA coding histone *H2B*-mCherry. To obtain information on birth or abortion, the blastocysts were transferred into pseudopregnant mice. The numbers of nuclei and their spatial positions were extracted, and the growth rate and shape of embryos in the “born” and “abort” cases were compared. Fluorescence observation facilitated data extraction and quantification. “Born” cases were those where the mouse fetuses survived to the end point of the experiment, while “abort” cases were those ending in spontaneous abortion. Moreover, to investigate whether the born/abort parameter also applies to human embryos, we performed a retrospective analysis using bright-field imaging movies of human embryos. In addition, immunostaining and live-cell imaging of mouse embryos were performed to investigate how the indicator affects embryogenesis.

In this study, we observed that early abortion is potentially caused by decreased ICM due to mitotic asynchrony, despite the presence of embryos with high synchrony among early abortion embryos. Approaches such as next generation sequencing to assess the involvement of the transcriptome and methylome are necessary to appropriately select embryos that will be born.

## Results

### Comparison between born and aborted embryos revealed the importance of synchrony in third mitosis

**Figure 1:**
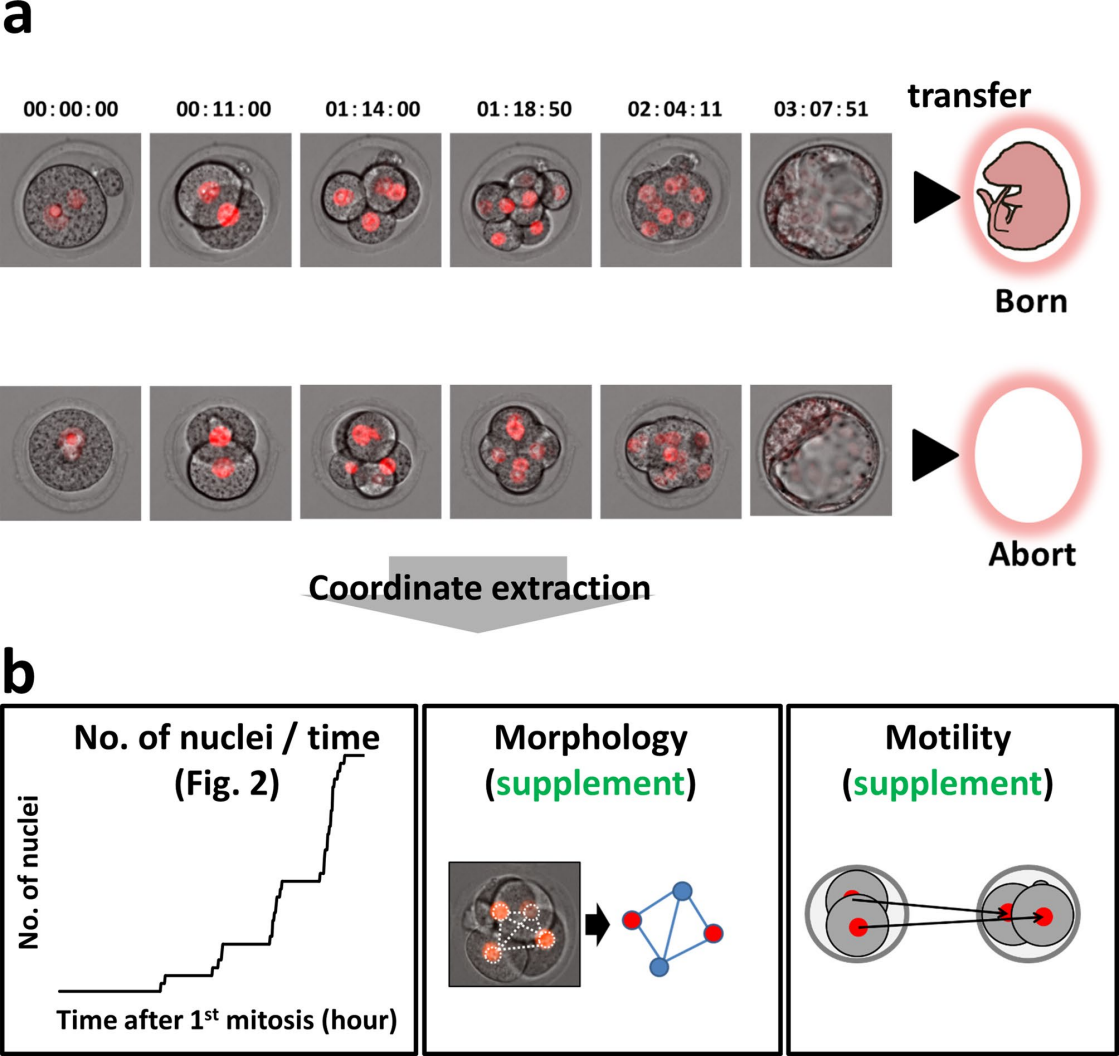
Comparative strategies and schemes for identifying indicators that predict embryo prognosis. (**a**) The zygote injected with the fluorescent probe histone *H2B*-mCherry mRNA was observed up to the blastocyst stage. Each blastocyst was transplanted into one pseudopregnant mouse, and the comparison was made retrospectively based on the transplantation results. (**b**) We measured the number of nuclei, morphology, and motility using barycentric coordinates.

**Figure 2:**
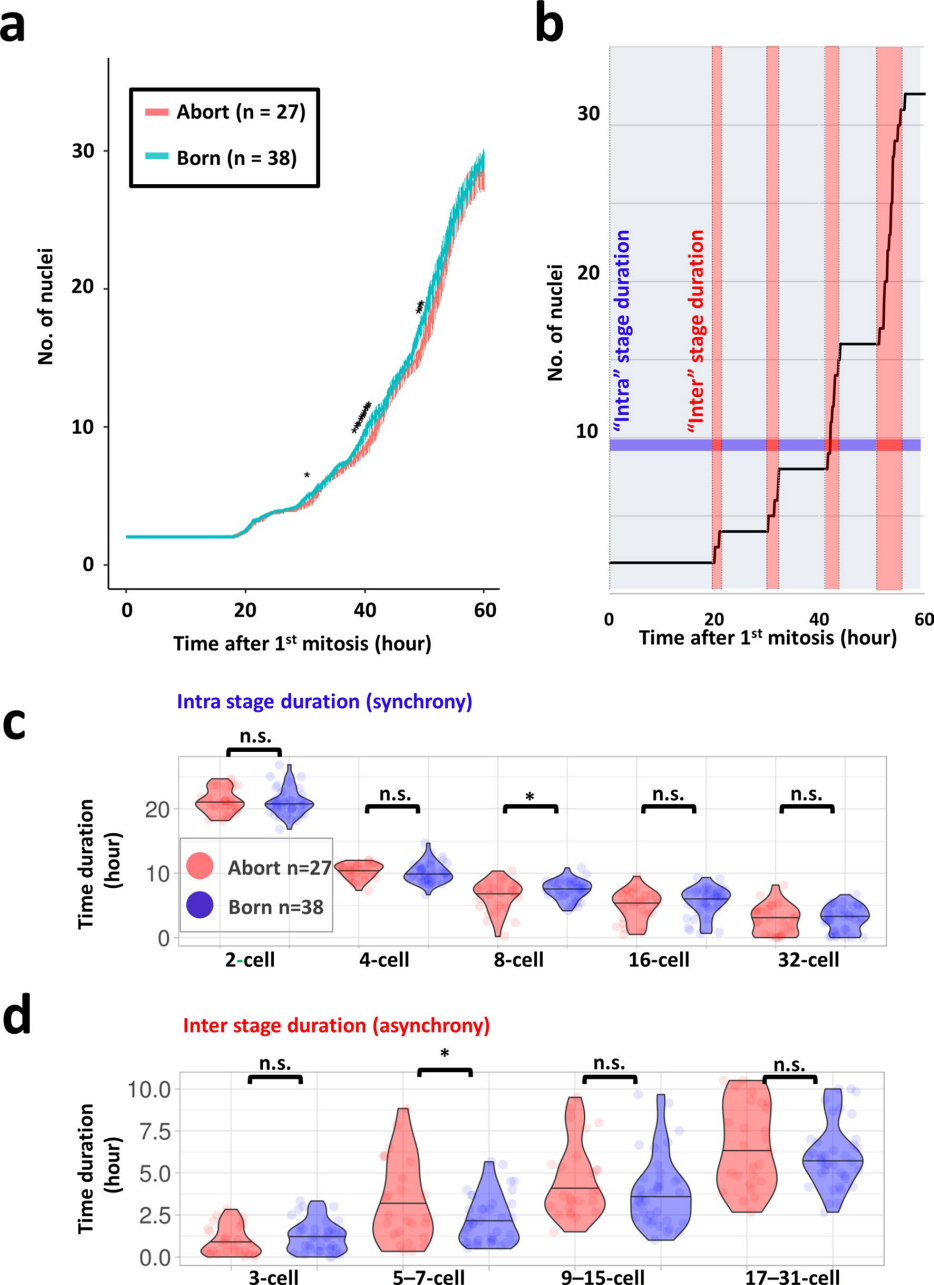
Comparison of cell number and time between born embryos and aborted embryos. (**a**) Comparison of averaged cell growth curves of born/abort embryos. *: significantly different. Data are represented as mean ± SD. We used 65 mouse zygotes for the retrospective study (we used 15 female and three male mice). (**b**) Schematic diagram of “Intra” stage duration and “Inter” stage duration. “Intra” stage denotes 2-, 4-, 8-, 16-, 32-cell stage duration, and Inter stage denotes 3-, 5–7-, 9–15-, 17–31-cell stage duration. When the cells were not dividing, shown in blue, the duration is the intra-stage duration. The time during which the cell was dividing, shown in red, represents the interstage duration. (**c**) Comparison of “intra” stage duration between born and abort embryos. (**d**) Comparison of “inter” stage duration between born and abort embryos.

Single ICR blastocysts were produced via routine IVF, and then, they were transferred into uteri 2 d post-coitum. Recipient females were sacrificed at D18.5 post-coitum to evaluate the full-term developmental ability of each analyzed embryo (born: alive at D18.5, abort: no sign of implantation). With live-cell imaging movies of mouse embryos using the *H2B*-mCherry probe^16^, the developmental pattern was compared between born (n = 38) and abort (no sign of implantation at D18.5; n = 27) case embryos (Fig. 1a). We intended to comprehensively evaluate the parameters that can be extracted and calculated from the fluorescence observation image of the nucleus. By creating a tool that can calculate the position coordinates from the fluorescent image, (See “Coordinate extraction” in Materials and methods) a comprehensive evaluation was achieved in this study. The morphology can be evaluated from the position information (coordinates). To easily evaluate the morphology, we adopted a method of calculating the coefficient of variation (CV) of the distance from each barycentric coordinates of the nuclei and a method of evaluating the deviation of the center of gravity arrangement (Procrustes analysis). In addition, the sum of the movement distances of cells per unit time (embryo motility) can be calculated from the position information and the time information. The barycentric coordinates of the nuclei were extracted (Supplemental Table 1 and Supplemental Table 2), and the number of nuclei, morphology (distance, coefficient of variation of distance, Procrustes analysis; Supplemental Figs. 1–5), and motility (Supplemental Fig. 6) were examined (Fig. 1b). There was no significant difference between born and abort embryos in terms of morphology and motility (Supplemental Figs. 1–6). Since the time at which in vitro fertilization (IVF) embryos were fertilized cannot be accurately estimated, the time when first mitosis occurred was taken as the starting point (t = 0). Comparison of the number of nuclei at different times revealed time points at which significant differences appeared periodically, at 30 h, 40 h, and 50 h after the first mitosis (Wilcoxon rank-sum test, *P* < 0.05; Fig. 2a). There was no significant difference in the time to 32 cells (two-sided Student’s t-test, *P* = 0.50) between born and abort groups. To identify the cell stage at which the difference was observed, the duration of 2-, 4-, 8-, 16-, and 32-cell embryo stages (intra-stage duration: when the cell was not dividing, the duration was the intra-stage duration.; Fig. 2b) and duration of 3-, 5–7-, 9–15-, and 17–31-cell embryo stages (inter-stage duration: the time during which the cell was dividing represented the inter-stage duration.; Fig. 2b) were assessed and compared between the born and abort cases. The duration of the 8-cell stage of abort embryos was shorter than that for born embryos (two-sided Welch’s t-test; *P* = 0.045, Fig. 2c). In addition, the duration for 5–7 cell stage of abort embryos was longer than that for born embryos (two-sided Welch’s t-test; *P* = 0.049: Fig. 2d). There was no significant difference in other durations.

**Figure 3:**
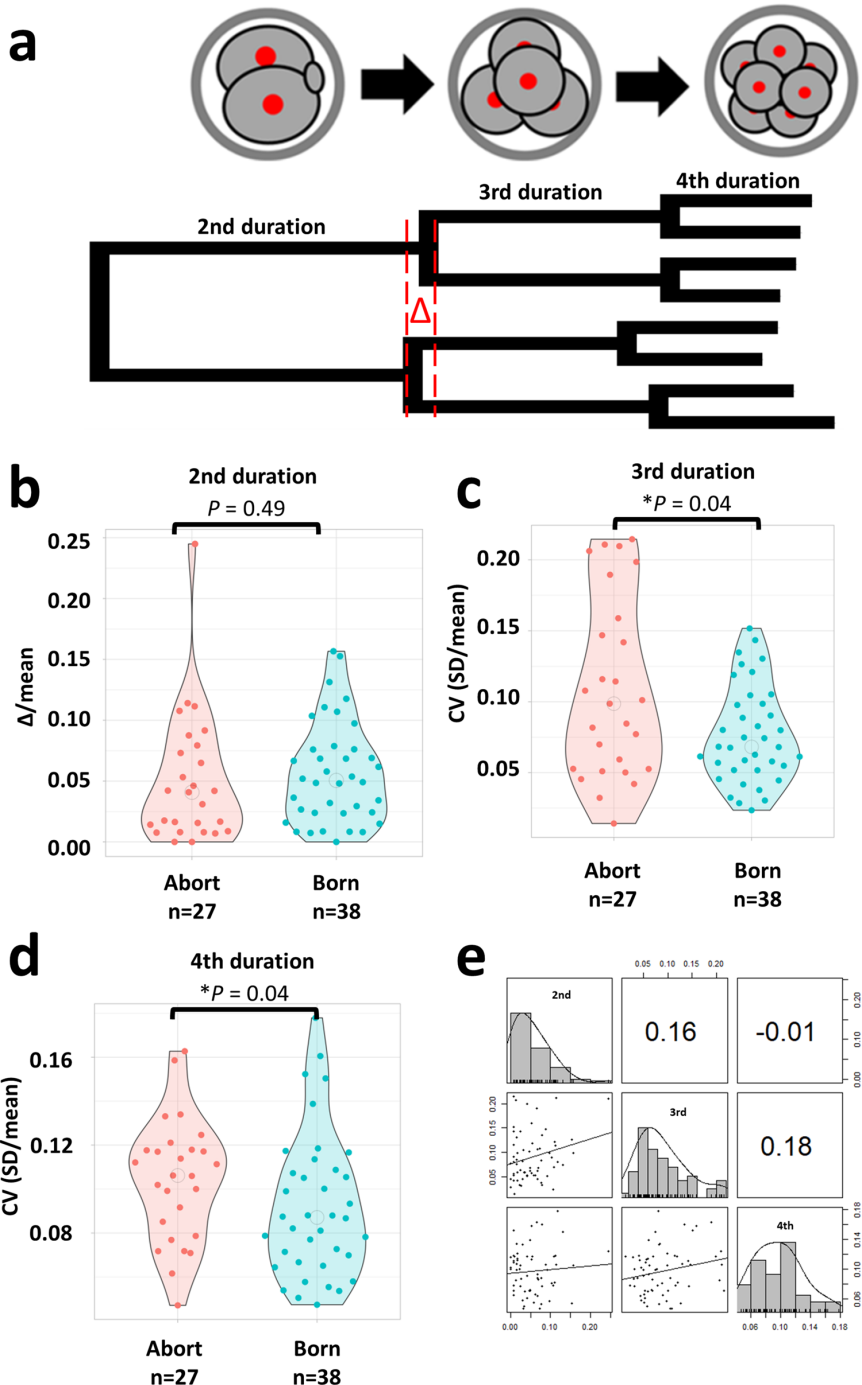
Variations in third and fourth duration are correlated with early abortion. (**a**) Tree diagram of the duration of stages of embryos. The red delta represents the difference between the duration of the second duration of each blastomere. (**b**) Comparison of variations in second duration blastomeres. Normalization was performed by dividing by the average time of second duration of each blastomere. We transferred 65 mouse zygotes for the retrospective study (we used 15 female and three male mice). Born: n = 38 Abort: n = 27. (**c**) Comparison of variations in third duration blastomeres. Born: n = 38 Abort: n = 27. Normalization was performed by dividing by the average time of third duration of each blastomere. (**d**) Comparison of variations in fourth duration blastomeres. Normalization was performed by dividing by the average time of fourth duration of each blastomere. Born: n = 38 Abort: n = 27. (**e**) Correlation of variations of second, third, and fourth duration. Correlation coefficients, histograms, and scatter plots are shown.

Subsequently, we investigated the relationship between the synchrony of mitotic stage and abort/born and their correlation. We measured the time taken for individual blastomeres to divide into 2–4 cells (second duration), 4–8 cells (third duration), and 8–16 cells (fourth duration) (Fig. 3a). The absolute value of the difference in the second duration was normalized by the mean value of each blastomere and compared between born cases and abort cases, but there were no significant differences (Fig. 3b; Wilcoxon rank-sum test, *P* = 0.49). The coefficient of variation (CV: standard deviation/average) of the duration of the blastomere during the third duration and the fourth duration in each embryo was measured and compared between born cases and abort cases. Abort embryos showed larger CVs of the third duration and fourth duration than born embryos (Fig. 3c,d; Wilcoxon rank-sum test, *P* = 0.04, *P* = 0.04). The second, third, and fourth asynchronization indicators were examined; however, no significant correlations were identified (Fig. 3e; Spearman’s rank correlation test, second vs. third: *P* = 0.20, third vs. fourth: *P* = 0.16, second vs. fourth: *P* = 0.93). Receiver operating characteristic (ROC) analysis was performed using the CV of the third period, as the embryos could be best separated by calculating the cutoff value (CV cutoff = 0.15, specificity = 1.0, sensitivity = 0.26, accuracy = 0.69). High-CV (CV ≧ 0.15) accounted for 25.9% (7/27) of abort cases and 10.7% (7/65) of all embryos.

**Figure 4:**
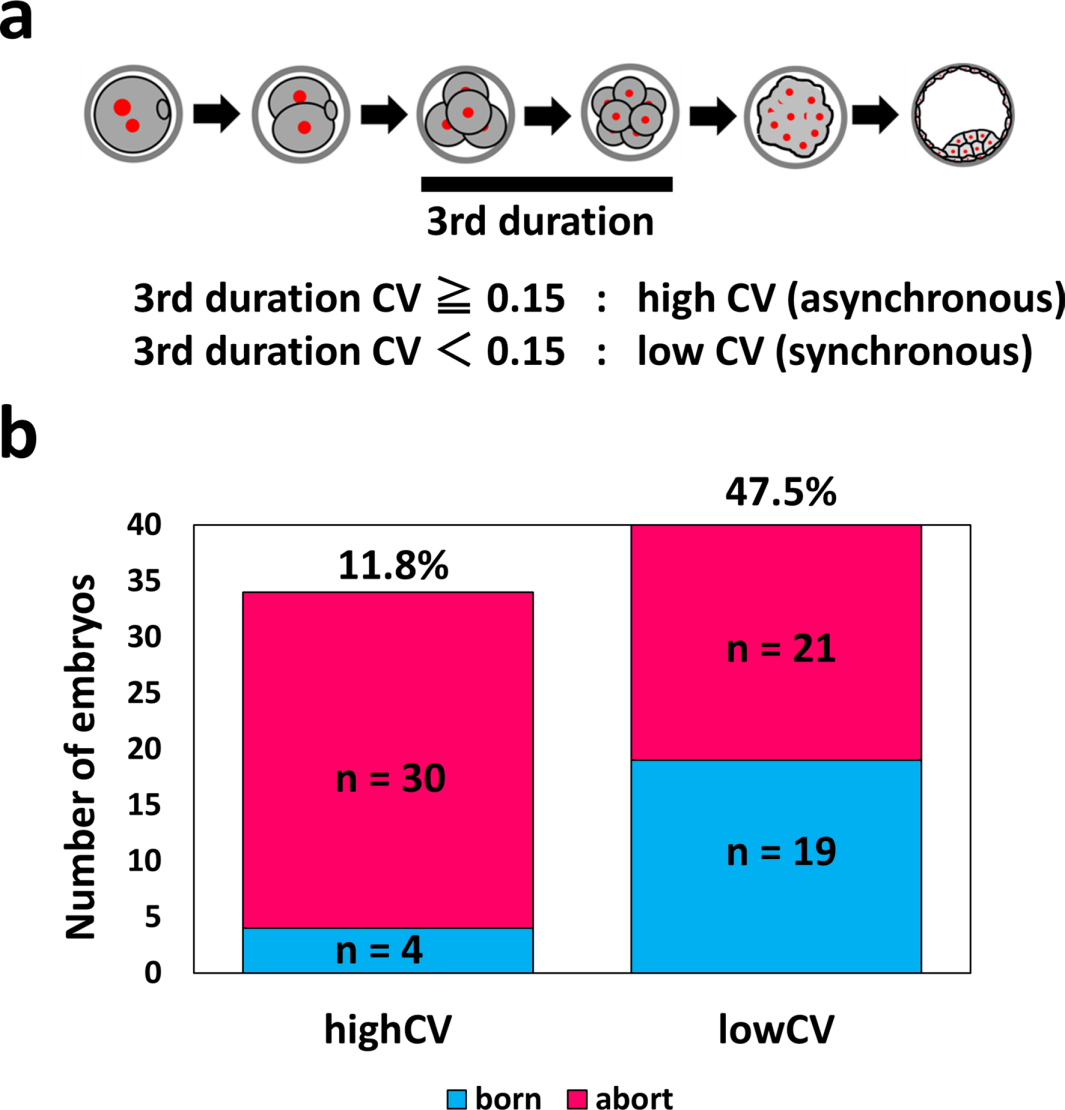
Prospective analysis by embryo selection and embryo transfer of asynchronous/synchronous embryos. (**a**) Scheme diagram for embryo selection. (**b**) Graph of transplantation results after embryo selection. For the prospective study, 74 mouse zygotes (we used 15 female and three male mice) were transferred. Born: n = 23 Abort: n = 51.

To perform prospective analysis for validating the effectiveness of the indicator, live-cell imaging was performed, and high-CV (asynchronous) embryos (n = 34) and low-CV (synchronous) embryos (n = 40) were transferred to the uterus of pseudopregnant mice (Fig. 4a; N [mouse] = 58). Following Cesarean section (If spontaneous delivery was adopted, some mothers may kill their offspring immediately after delivery. As this could lead to an inaccurate assessment of the number of offspring, we opted to perform Caesarian section), the born rates of high- and low-CV blastocysts were 11.8% (4/34) and 47.5% (19/40), which were significantly different (Fig. 4b; prop-test, P = 0.002).

### Human early aborted embryo also shows asynchronous division

**Figure 5:**
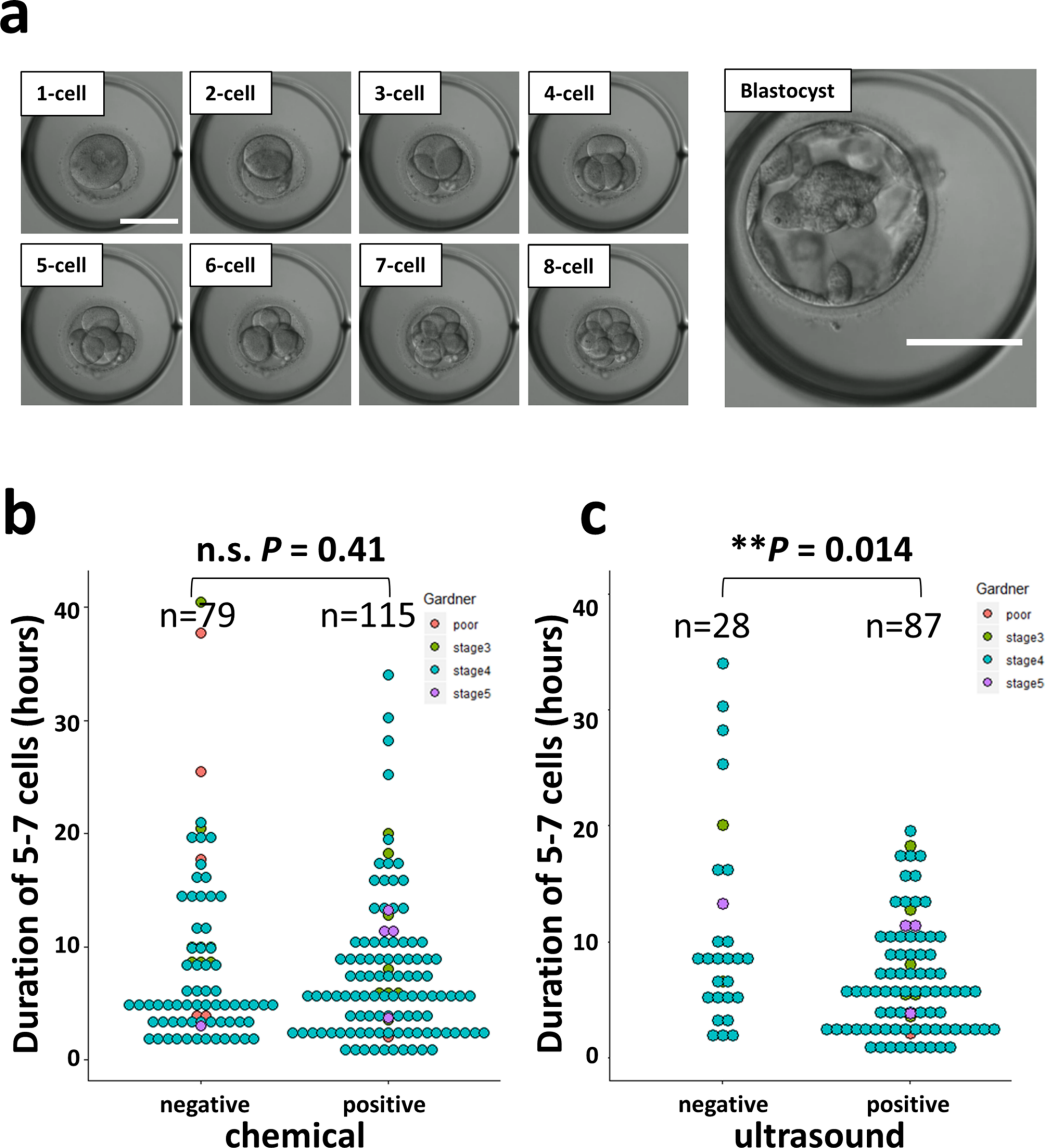
Bright-field observation of human embryos reveals that the third division is related to early abortion. (**a**) Stills taken from movies of human embryos getting infertility treatment were retrospectively analyzed to calculate the time to the 8-cell stage. The embryos growing up to the blastocyst stage were observed and scored by Gardner classification. Bar = 100 µm. In this cohort, 194 human zygotes were analyzed. (**b**) Comparison of duration of 5–7 cells between positive (n = 115) and negative (n = 79) groups of chemical pregnancy tests. (c) Comparison of duration of 5–7 cells between ultrasound-positive (n = 87) and negative (n = 28) groups.

To investigate whether the high CV in the 3rd duration was observed in human embryos as well, the development of human embryos was observed using bright-field imaging and prognosis after transplantation (or progress of pregnancy) during infertility treatment (Fig. 5a). We calculated the duration of each stage from the bright-field movie taken during infertility treatment and investigated the relationship with the human chorionic gonadotropin (hCG) test, which is an index of implantation, or the echo-test, which is an index of subsequent development. Since it was a bright-field observation, it was difficult to calculate the division time after the 8-cell stage. Therefore, the time up to the 5–7-cell stage was measured, and the correlations between the duration of the 5–7-cell stage and the chemical pregnancy confirmation (hCG positive/negative) or transvaginal ultrasound scan were confirmed. We analyzed one cohort as the difference of successful implantation (early: hCG positive/negative) and embryo growth (late: transvaginal ultrasound scan), respectively. There was no significant difference in the length of the 5–7 cell stage between the negative (n = 79) and positive groups (n = 115) of chemical pregnancy (*P* = 0.41, two-sided Student’s t-test. Figure 5b). However, there was a significant difference in the length of the 5–7 cell stage between the ultrasound-negative group (n = 28) and the ultrasound-positive group (n = 87, *P* = 0.014, two-sided Welch’s t-test. Figure 5c). When applying Gardner criteria, embryos with < 3BB (an indicator of poor embryo prognosis, as seen in Teranishi et al.^17^ did not exist in the ultrasound-negative group, suggesting that asynchrony did not reflect the Gardner classification. There was no significant difference in age between the two groups (Wilcoxon rank-sum test, *P* = 0.32, +: average 33.5 years old vs. −: average 33.8 years old). It seemed that four cases with an exceptionally long duration induced the statistical difference. Upon excluding these four cases, no significant difference (*P* = 0.16, t-test) was observed. This suggests that asynchrony is a risk factor that accounts for 14% (4/28) of ultra-sound negatives. The ages of these four cases were 32, 34, 35, and 37 years, respectively, and no significant difference (*P* = 0.55, n = 4 vs. n = 24 t-test) was observed in the ultrasound-negative group, except for these four cases. This suggested an abortion risk associated with transplanting embryos with durations of 20–30 h or more, which cannot be estimated by age.

### Asynchrony of the third duration leads to a decrease in ICMs

**Figure 6:**
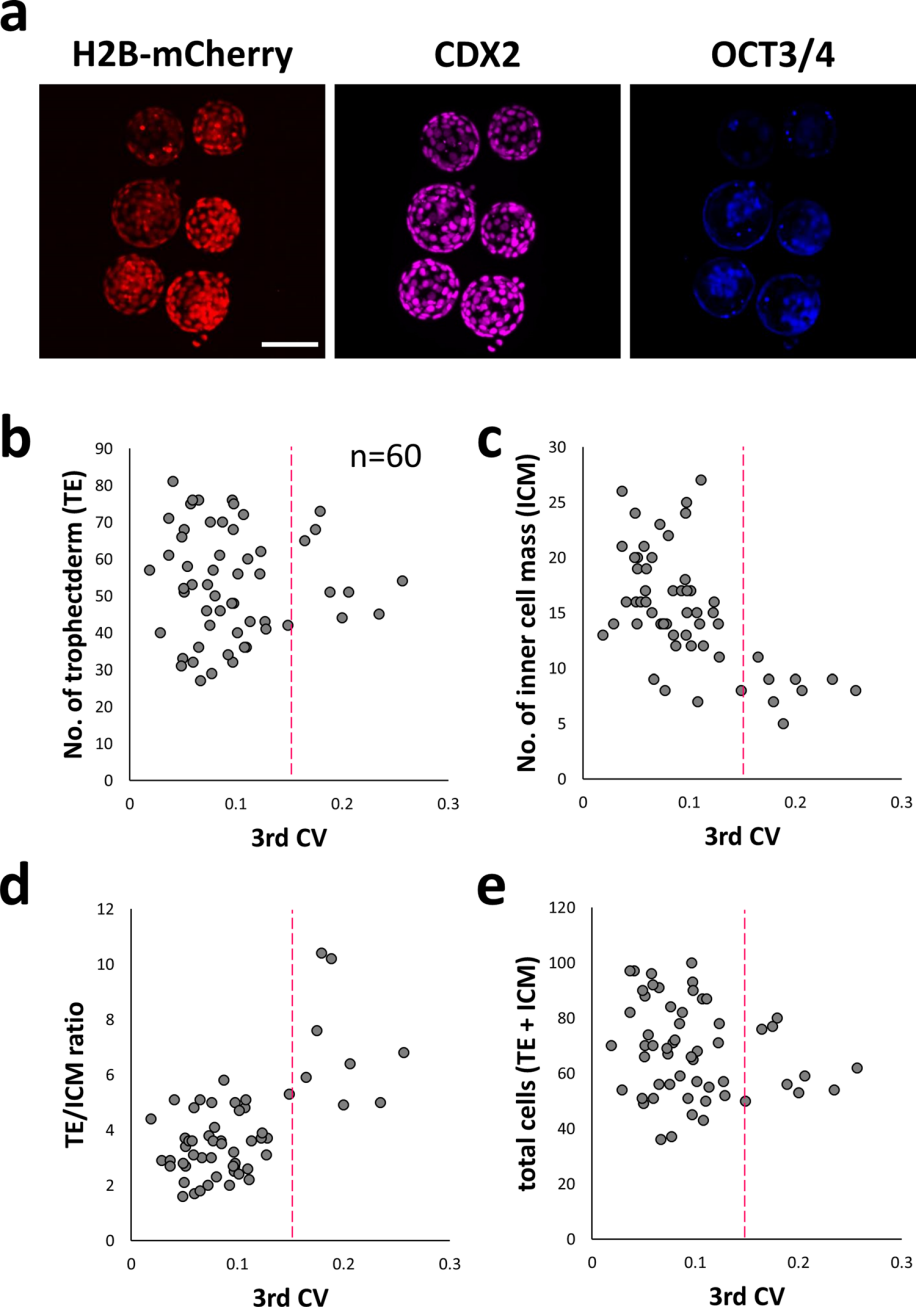
Relationships between synchrony and number of cells of ICM/TE. (**a**) Fluorescence images of H2B-mCherry-expressing, CDX immunostained, and OCT3/4 immunostained embryos. Bar = 100 µm For this analysis, 60 mouse zygotes (we used 15 female and three male mice) were observed. (**b**) Correlation between third CV and number of TE. (**c**) Correlation between third CV and number of ICM. (**d**) Correlation between third CV and TE/ICM ratio. (**e**) Correlation between third CV and total number of cells (TE + ICM).

The number of ICM cells and total cells of the blastocyst are factors that affect the birth rate^18^. After the CV of the third duration was calculated (n = 56), ICM and TE were immunostained using OCT3/4 (ICM marker^19^) and CDX2 (TE marker^20^) antibodies, and three-dimensional (3D) images were taken with a confocal microscope. (Fig. 6a, maximum intensity projection images are shown). Using 3D images, the number of ICM/TE cells was counted. No correlation was found between third CV and TE cells (Fig. 6b, Spearman’s rank correlation test, *P* = 0.6), but significant correlation was observed between the CV of the third duration and ICM cells (Fig. 6c, Spearman’s rank correlation test, *P* = 1.0 × 10^−5^). The third CV and the TE/ICM ratio also showed a significant correlation (Fig. 6d, Spearman’s rank correlation test, *P* = 3.8 × 10^−4^). There was no significant correlation between third CV and total cells (Fig. 6e, Spearman’s rank correlation test, *P* = 0.1). In addition, we found a lower number of inner cells in the high CV group (high CV: 8.3 ± 1.8, low CV: 16.3 ± 4.6) following injection with histone H2B-mCherry mRNA (see Supplemental Fig. 7).

### Asynchrony of third duration causes delay in compaction in some cells and changes the time of YAP1 nuclear localization

**Figure 7:**
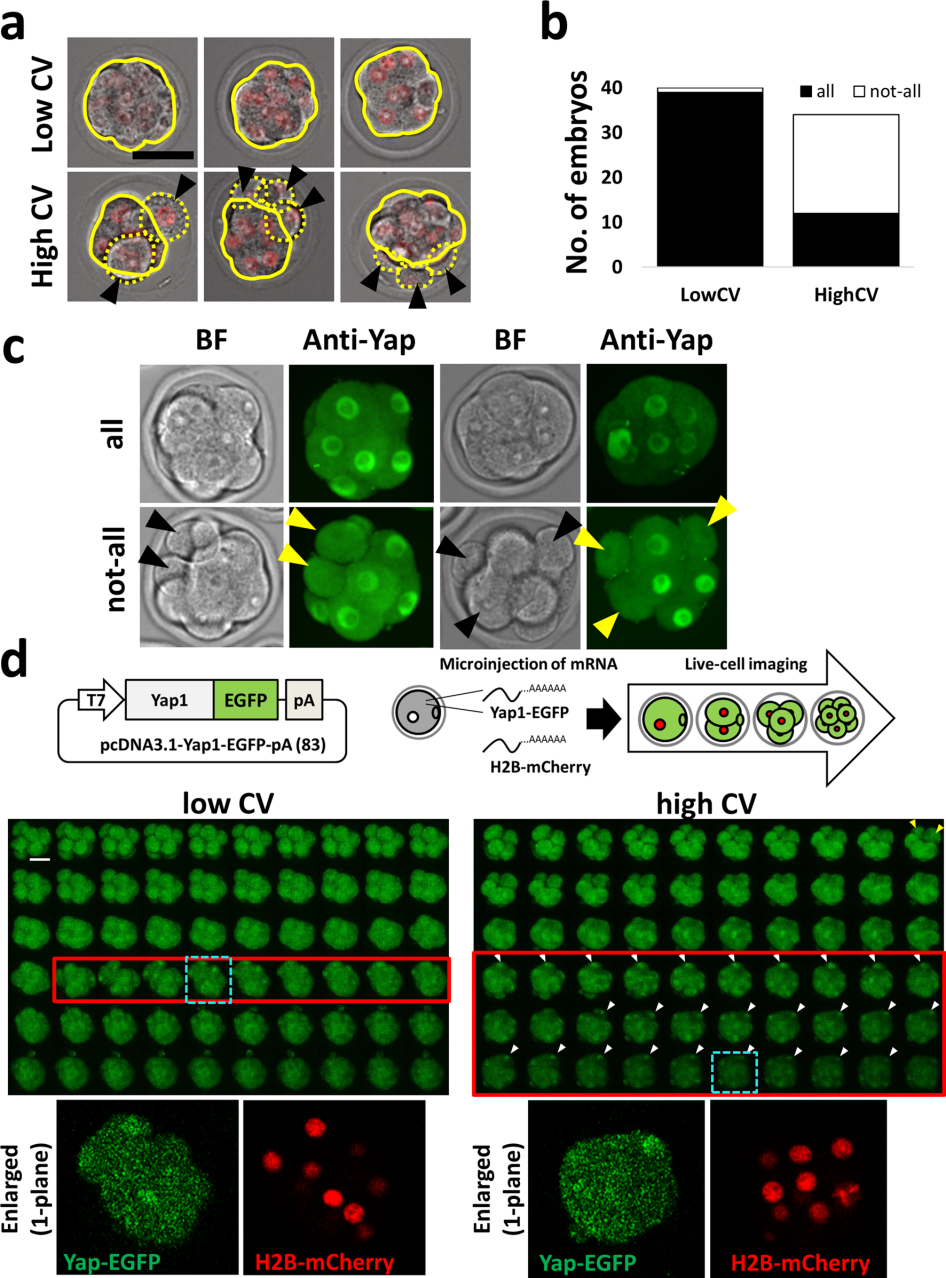
Third division synchrony affects compaction and YAP1 localization. (**a**) Comparison of the state of compaction between an embryo showing a high CV and an embryo showing a low CV. Black arrowheads represent blastomeres that did not become flattened. Bar = 50 µm. (**b**) Relationship between not-all/all cells became flattened and low CV/high CV. For this analysis, 74 mouse zygotes (we used 15 female and three male mice) were observed. (**c**) Not-all/all flattened embryos immunostained with the anti-YAP1 antibody. Black and yellow arrowheads represent blastomeres that do not contribute to compaction. Bar = 50 µm. We used mouse 56 zygotes for immunostaining (we used five female and one male mice). (**d**) YAP1 localization by live-cell imaging using *Yap1*-EGFP mRNA. The upper left illustration shows the plasmid that we used. The upper right illustration shows the scheme of the experiments. The middle left panel shows the embryo with low CV at third division. Video is at 10 min intervals. Red frames show the durations in which the YAP1-EGFP signal was strongly seen in the nucleus. The middle right panel shows the embryo with high CV at third division. Yellow arrowheads indicate blastomeres that do not contribute to compaction at this point, and white arrowheads indicate blastomeres derived from blastomeres shown with yellow arrowheads, wherein Yap1-EGFP was localized to the nucleus. The bottom panels show an enlarged single-plane image. Bar = 50 µm. We used 49 zygotes for the observation of YAP1-EGFP (we used five female and one male mice).

Retrospective analysis to investigate the relationship between third division synchrony and compaction revealed that most embryos in the low CV (synchrony) group (39/40) were flattened at the same time as the flattening of all blastomeres (It was judged visually). In the high CV (asynchronous) group, most blastomeres (22/34) did not flatten at the same time (not all the blastomeres became flattened; Fig. 7a,b, Supplemental Movie 1). As far as we observed, this is not due to de-compaction coupling to mitotic cells^21^ but due to delay of compaction in part of the cells (Supplemental Movie 1). Using live-cell imaging, we observed that this phenomenon is not simply derived from dividing cells (Supplemental Fig. 8). Nuclear localization of Yes-associated protein-1 (YAP1) is controlled by mechanotransduction processes such as contractility and influences the fate of TE^22–24^. YAP1 was observed by immunostaining, with no nuclear YAP1 observed in blastomeres that were not compacted (Fig. 7c). Since the subsequent localization of YAP1 in fixed cells is not known, a *Yap1*-EGFP expression vector was prepared, mRNA was injected into fertilized eggs, and the change in localization was observed by live-cell imaging. We used 5 ng/µL of *Yap1-*EGFP mRNA because this concentration does not affect the subsequent localization of CDX2^24^. We investigated whether the nuclear translocation of Yap1 in cells will be delayed. If the nuclear translocation is delayed, the total time for localizing Yap1-EGFP in the nucleus will be longer than that of the synchronous embryo. We found that blastomeres with delayed compaction had a longer duration where Yap1 was localized in the nucleus [low CV (n = 9): 8.8 ± 2.7 h vs. high CV (n = 3): 17.6 ± 2.8 h, *P* = 0.009, Wilcoxon rank-sum test, Fig. 7d].

## Discussion

Comprehensive analysis and evaluation of the parameters that can be extracted from images and videos will be crucial in embryo evaluation research. In the present study, by comprehensive analysis, we found that asynchrony at the third division is a risk factor for abortion, based on not only retrospective analysis but also prospective analysis. We also confirmed that asynchrony at the third division is a risk factor for early abortion in human embryos. In mouse embryos, asynchrony at the third division caused a decrease in ICM cells. Furthermore, we found that YAP1’s nuclear localization time was delayed due to the slower compaction in asynchronous blastomeres.

Motato et al.^25^ found that the embryos showing a higher t5–t8 (time to reach 5-cell − time to reach 8-cell) showed a lower implantation ratio. This result is consistent with our result that the chemical pregnancy-positive (hCG-positive) and ultrasound-negative (early aborted) groups had a long 5–7-cell duration. Furthermore, Hlinka et al.^26^ argue that blastocysts that have undergone untimely division including 4- to 8-cell asynchrony have an extremely low pregnancy rate. However, past morphokinetic studies were concluded only by retrospective analysis of human embryos and hence were limited in their scope. We performed both prospective and retrospective analyzes using mouse embryos and confirmed that asynchrony is the risk factor for abortion.

An event that occurs in the vicinity of the third division that can influence the differentiation of ICM/TE is compaction, which is cell flattening and close intercellular apposition during the transition from 8 to 16 cells^27^. Lagalla et al.^28^ showed that irregularly cleaved embryos have a higher proportion of not-all of cells compacted morulae than normally cleaved embryos. Asynchrony at third division is likely due to the expression fluctuation of each blastomere in the 2- or 4-cell stage. Li et al.^29^ showed that injection of *Aurora kinase B* (*AurkB)* mRNA into one blastomere at the two-cell stage increased time from nuclear envelope breakdown to sister chromatid separation and decreased OCT4-positive cells, while overexpressed blastomeres were more likely to contribute to the placenta. The effects of injecting *AurkB* mRNA are similar to the results obtained in this study in that they affect the time of division and cause a decrease of Oct3/4-positive cells. Thus, fluctuations in the expression of proteins involved in the cell cycle may cause the observed phenomena. Korotkevich et al.^30^ revealed that a few remaining apolar daughter cells were repositioned to the embryo surface and acquired an apical domain, and most of these cells eventually turned on *Cdx2* expression, albeit later than other cells. Based on the findings in existing reports and those of our study, we suggest that synchrony may be delayed due to stochastic variation in gene expression, particularly variation in genes related to the cell cycle; the timing of compaction is delayed, and cells with lesser polarity appear, which contribute to TE. The rest of the cells determine ICM/TE differentiation by a tug-of-war between polarity and shape^31^, but the low population of these cells may reduce the number of ICMs. However, there was no significant difference in TE and total cells in our ICM and TE counts. When power analysis was performed, n = 7 in each group was sufficient for analyzing the ICM, but when testing for TE and total cells, a significant difference could be inferred by comparing 56 in each group. In this paper, we emphasize that at least asynchrony was related to the low number of ICMs and the different expression patterns of YAP. Of course, the hypothesis discussed in this section should be confirmed based on methods different from ours (e.g. knock-in of YAP-EGFP into an intrinsic locus) in the future.

For selection of human embryos, the shape of the blastocyst is important^5^. In the present study, most of the embryos with delayed 5–7-cell stage and abortion had good morphology with stage 4 according to the Gardner classification (Fig. 5). Therefore, the blastocyst shape and synchrony at the third division are independent indicators of reduced risk of early abortion.

We analyzed the video of human embryos, but as the video recording from fertility treatment was taken on a single plane, we could not observe embryos three-dimensionally. Therefore, it was difficult to calculate the duration of the 8-cell stage, the time per blastomere, and the CV. Three-dimensional bright-field observation could overcome this problem. Because the time schedules of events during preimplantation-like genome activation and compaction are different between humans and mice, this study had limitations other than mentioning the importance of division synchrony in early division. If the number of cells can be freely counted in the bright field, it will be possible to perform an analysis considering the time schedule.

We suggest that early abortion was caused by the decrease in ICM due to mitotic asynchrony. However, there were embryos with high synchrony among the early abortion embryos. Therefore, this indicator cannot detect all cases of early abortion. In the future, we expect to be able to appropriately select embryos that will be born by investigating the remaining causes with new approaches such as next generation sequencing to assess involvement of the transcriptome and methylome. In addition, an attempt to comprehensively analyze and evaluate the parameters that can be extracted from images and videos (other than this research, there was an attempt by deep learning^32^) will also be important for embryo evaluation research.

## Materials and methods

### Animals

The reporting in the manuscript followed the recommendations in the ARRIVE guidelines. This study conformed to the Guide for the Care and Use of Laboratory Animals. All animal experiments were approved by the Animal Care and Use Committee at the Research Institute for Kindai University (permit number: KABT-31–016). ICR strain mice (male and female, 12–16 weeks old, wild type, healthy mice, no involvement in previous procedures) were obtained from Japan SLC, Inc. (Shizuoka, Japan). Room conditions were standardized, with the temperature maintained at 23 °C, relative humidity at 50%, and a 12 h/12 h light–dark cycle. Animals had free access to water and commercial food pellets. Mice used for experiments were sacrificed by cervical dislocation.

### In vitro fertilization (IVF)

IVF was performed as described previously^14^. Briefly, females were superovulated by injecting 10 IU of pregnant mare serum gonadotropin (ASKA Pharmaceutical Co., Ltd., Tokyo, Japan) to bring the oocytes to maturity. This was followed by administering 10 IU of human chorionic gonadotropin (ASKA Pharmaceutical Co.) 48 h later to release the oocytes. Ovulated oocytes were collected from the oviducts 14 h after injection. Cumulus-enclosed oocytes were placed in 200 µL drops (2 drops) of TYH medium^33^ and covered with liquid paraffin (Nacalai Tesque, Kyoto, Japan). Spermatozoa were collected by mechanically dissecting the cauda epididymites and were placed in 200 µL drops of TYH medium. After 2 h of incubation, spermatozoa gained the ability to fertilize, and the sperm suspension was added to the TYH drops containing eggs at a concentration of 100 sperm/μL and incubated for 2 h at 37 °C under 6% CO_2_ in air to allow spermatozoa to fertilize the oocytes. Cumulus cells were dispersed by brief treatment with hyaluronidase (Type-IS, 150 U/mL, Sigma, St. Louis, MO, USA). Three hours after the dispersion of cumulus cells, the number of pronuclei was counted to check for fertilization.

### Ethics statement

This study performed in accordance with the Declaration of Helsinki.

Human participants' names and other HIPAA identifiers were removed from all sections of the manuscript, including supplementary information. Analysis of human embryo movies was carried out with the approval of the Ethics Committee of Asada Ladies Clinic (Approval number: 2021-04), Kindai University (approval number: R2-1-001), and the Japanese Society of Obstetrics and Gynecology (date of approval: 06/30/2021). Informed consent was obtained from all patients included in the study.

### Plasmid construction

The DNA sequence of KpnI (GGTACC)—mouse *Yap1*—(Linker: GATCCACCGGTCGCCACC)-EGFP—NotI (GCGGCCGC) was synthesized by Eurofins Genomics. Synthesized DNA was digested by KpnI (R0142, New England BioLabs) and NotI (R3189, New England BioLabs), and was inserted into multi-cloning site of pcDNA3.1 vector.

### Live-cell imaging

The method used to prepare mRNA encoding histone *H2B*-mCherry was described previously^34^. Briefly, mRNA was prepared with the RiboMAX™ Large-Scale RNA Production Systems-T7 (Promega, Madison, WI, USA). The 5′ end of mRNA was capped using a Ribo m7G Cap Analog kit (Promega) to prevent degradation. Synthesized RNA was purified by phenol–chloroform treatment and subsequent gel filtration using a MicroSpin™S-200 HR column (GE Healthcare, Amersham, UK) to remove reaction intermediates and then stored at − 80 °C until use. *H2B*-mCherry mRNA (5 ng/μL) was injected into the cytosol of the zygote at the pronuclear stage using a piezo manipulator in HEPES-buffered Chatot–Ziomek–Bavister (CZB) medium^35^. The injected zygotes were transferred into 5 μL drops of KSOMaa medium^36,37^ containing 0.00025% polyvinyl alcohol and 100 µM EDTA on a film-bottom dish (Matsunami Glass Ind., Ltd., Osaka, Japan). Embryos were imaged three-dimensionally using a boxed type confocal laser microscope with an incubation chamber (CV1000, Yokogawa Electric Corp., Tokyo, Japan) set at 37 °C in 6% CO_2_, 5% O_2_, and 89% N_2_ with saturated humidity. We performed live-cell imaging four times. The recorded video was analyzed by Fiji^38^.

### Embryo transfer

Mouse embryo transfer was performed as described previously^16^. Briefly, single ICR blastocysts were produced by routine IVF, then were transferred into uteri 2 d post-coitum. To avoid natural births, recipient females were sacrificed 18.5 d post-coitum to evaluate the full-term developmental ability of each analyzed embryo.

### Immunostaining

Embryos were fixed at 20℃ in 4% paraformaldehyde and 0.1% polyvinyl alcohol in PBS for 30 min, permeabilized in 0.25% Triton-X 100 in PBS for 20 min, and blocked in 3% bovine serum albumin in PBS for 1 h. Mouse monoclonal anti-CDX2 (1:500, overnight, MU392-UC, BioGenex, San Ramon, CA), rabbit polyclonal anti-OCT3/4 (1:500, o/n, sc-9081, Santa Cruz Biotechnology, Inc., Dallas, TX), and rabbit polyclonal anti-YAP1 (1:100, o/n, #14074, Cell Signaling Technology, Danvers, MA) were used as primary antibodies. Alexa Fluor–conjugated secondary antibodies (1:500; 1 h; A32728, ab175661, Abcam) were used. Laser scanning confocal images were acquired by using a CSU-W1 SoRa microscope (Yokogawa Electric Corp., Tokyo, Japan).

### Morphometrics

The Procrustes analysis^39^ was performed using the R package shapes (version number: 1.2.5, URL: https://cran.r-project.org/web/packages/shapes/). The R package corrplot (URL: https://cran.r-project.org/web/packages/corrplot/) was used to create the Procrustes distance map.

### Coordinate extraction

From the stacked images of multiple embryos observed simultaneously, a 4D stacked image of each embryo was extracted by manually specifying the region of interest. For each time slice of the 4D stacked image, we applied the following 3D image processing to identify nuclei centers in the embryo:

1. Nuclei signal in the 3D volumetric image was amplified by subtracting background signal, applying histogram equalization, and removing noise.
2. The bias in the intensity along the z-axis was adjusted.
3. A binary mask for the whole nuclei region was calculated by thresholding and removing of small connected components.
4. The binary mask was subdivided into a set of smaller submasks such that each submask could cover one nucleus by repeating the following procedure; For each candidate of the submask, its length and width were measured. If one of those values was greater than a specified threshold calculated from the medians of those values of all the submasks, then the submask was subjected to subdivision; the subdivision was conducted by the watershed algorithm. This procedure stopped either when no candidate submask remained or when the stipulated number of iterations was conducted.
5. Finally, we measured the properties of each submask of the nucleus, e.g., centroid and volumetric size. The details and parameter values of each process are shown in a sample code provided at https://github.com/Q-bio-at-IIS/mashiko_2022_Sci_Rep. The centroids of nuclei obtained automatically by the procedure above contained various errors such as false detections of noisy signal as nuclei or failures in detecting existing nuclei.

We corrected these errors manually by comparing the automatically detected centroids with the embryo's volume-rendered 3D images. All programs were coded and executed on Mathematica version 10 (https://www.wolfram.com). The original xyz resolution was x: 0.8 µm/pixel, y: 0.8 µm/pixel, z: 2.0 µm/slice, but since it was set to 1: 1: 1 by internal processing, the extracted z coordinate must be divided by 2.5.

### Statistics and reproducibility

Normality was tested with Kolmogorov–Smirnov test; variability was tested with F-test; two groups were tested with Student’s t-test, Welch’s t-test, and Wilcoxon rank-sum test. Proportions were tested with prop-test. Correlations were determined with Spearman’s rank correlation test. These tests were performed using R (https://www.r-project.org/). We used 65 mouse zygotes for the retrospective study (we used 15 female and 3 male mice). For prospective study, 74 mouse zygotes were used (we used 15 female and 3 male mice). We analyzed 194 human embryos for the retrospective study. We used mouse 56 zygotes for immunostaining (we used five female and one male mice). We used 49 zygotes for observation of YAP1-EGFP (we used five female and one male mice).

### Video analysis of human embryos

The videos of the human embryos used in this study were taken using the zygote observation system (CCM-iBIS NEXT, ASTEC Co., Ltd., Fukuoka, Japan) at the Asada Ladies Clinic. The captured video was analyzed using VSDC free video editor (http://www.videosoftdev.com/jp).

## Supplementary Information

Supplementary Information 1.

Supplementary Video 1.

Supplementary Information 2.

Supplementary Information 3.

Supplementary Information 4.

## Data Availability

Dataset of coordinates was uploaded as Supplemental Table 1. The R code that we used to analyze morphology and motility has been uploaded to GitHub (https://github.com/mashikodaisuke/Supplemental_code/tree/main/code_supplement). The movies and coordinate extraction code supporting the current study have not been deposited in a public repository because we are still analyzing them for another study, but they are available from the corresponding author on request.
