# Supplementary material for: Asynchronous division at 4–8-cell stage of preimplantation embryos affects live birth through ICM/TE differentiation
Source: Sci Rep. 2022 Jun 7;12:9411. doi: 10.1038/s41598-022-13646-8 (PMC9174281; doi:10.1038/s41598-022-13646-8)
Supplement: Supplementary file 1 — Supplementary Information 1. [file 41598_2022_13646_MOESM1_ESM.pptx]

## Slide 1
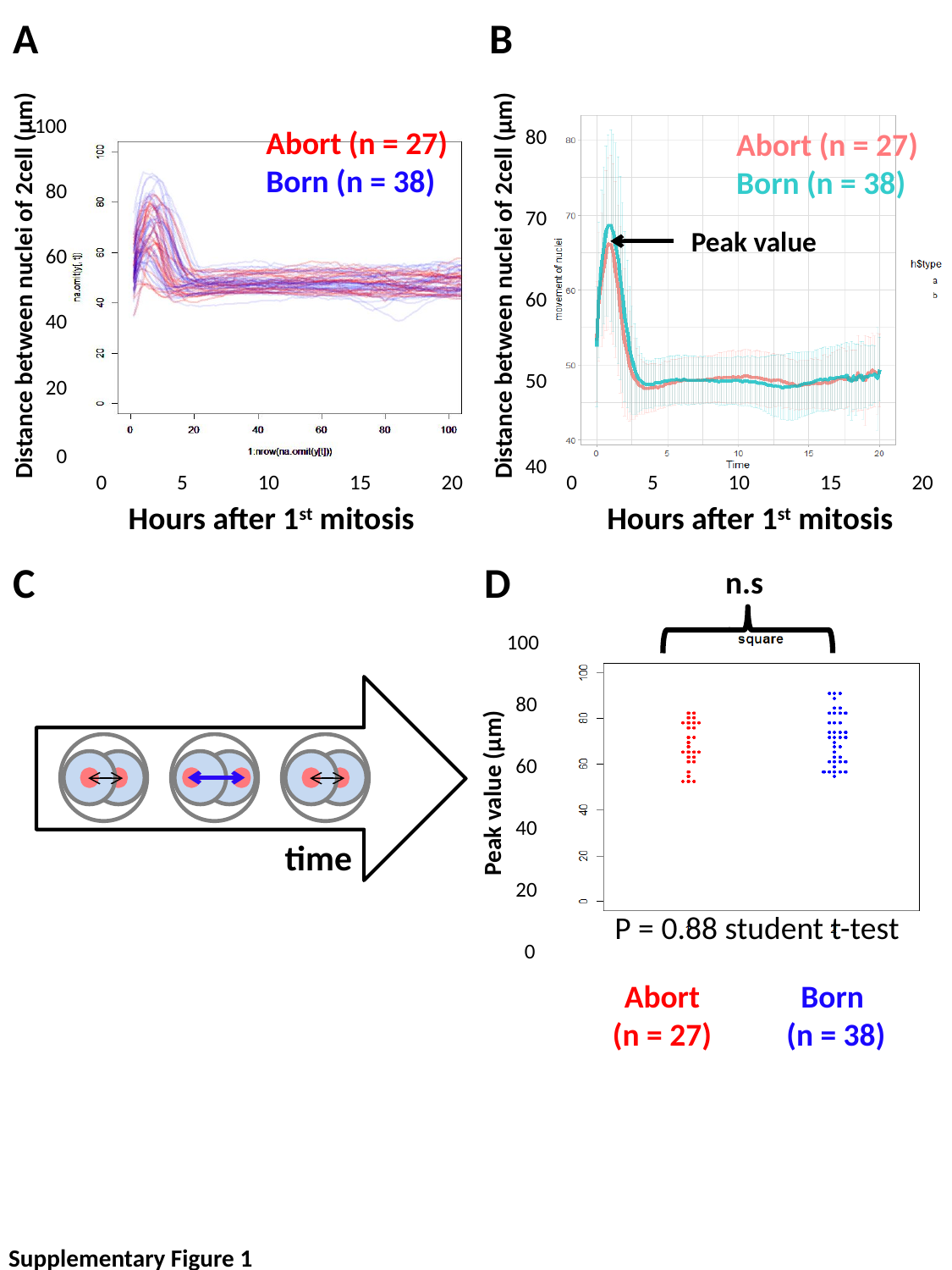

A
B
100
Abort (n = 27)
Born (n = 38)
80
Abort (n = 27)
Born (n = 38)
80
70
Peak value
60
Distance between nuclei of 2cell (µm)
Distance between nuclei of 2cell (µm)
60
40
50
20
0
40
0
5
10
15
20
0
5
10
15
20
Hours after 1st mitosis
Hours after 1st mitosis
C
D
n.s.
100
80
60
Peak value (µm)
40
time
20
P = 0.88 student t-test
0
Abort
 (n = 27)
Born
(n = 38)
Supplementary Figure 1

## Slide 2
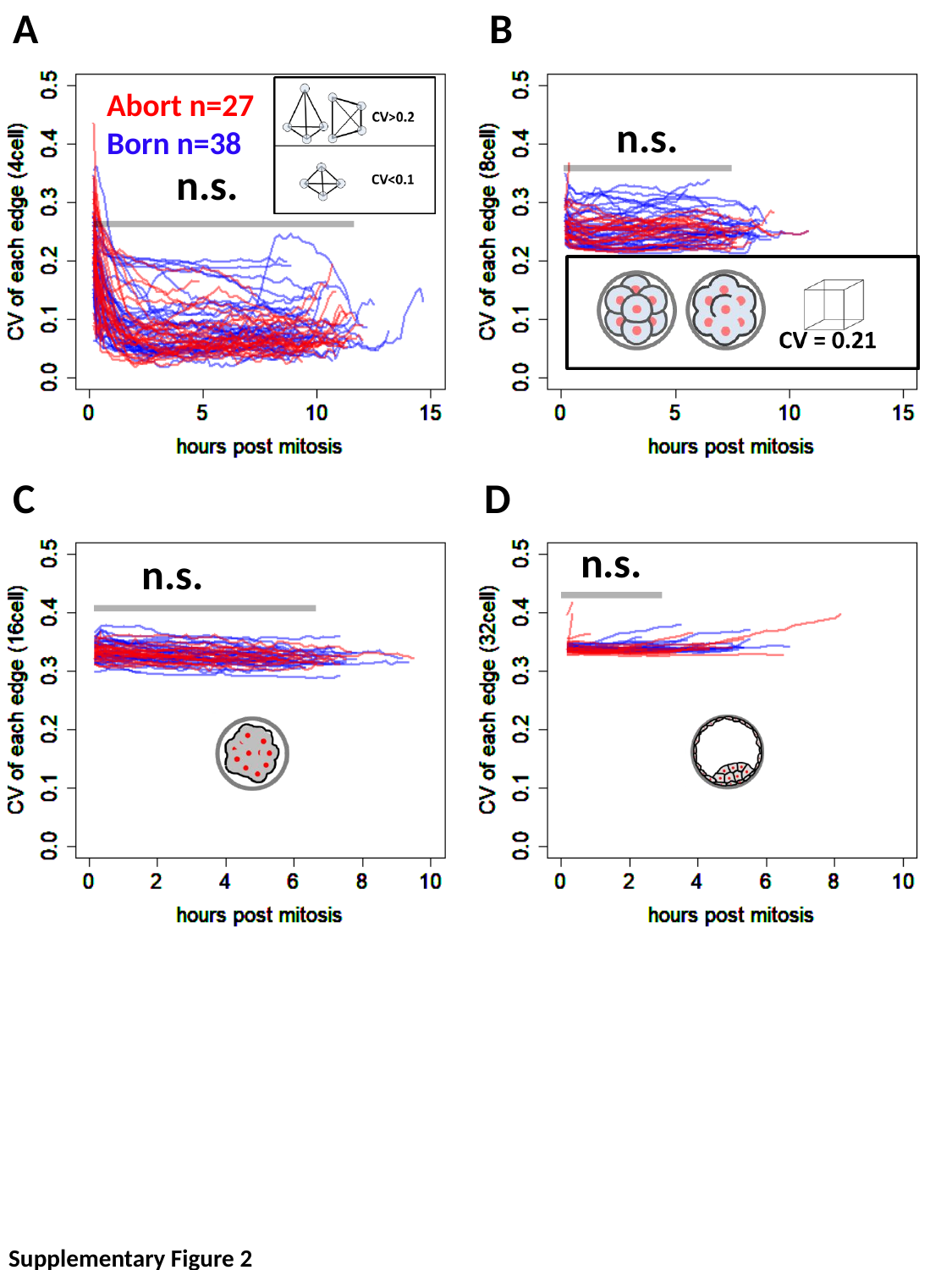

A
B
Abort n=27
Born n=38
n.s.
n.s.
C
D
n.s.
n.s.
Supplementary Figure 2

## Slide 3
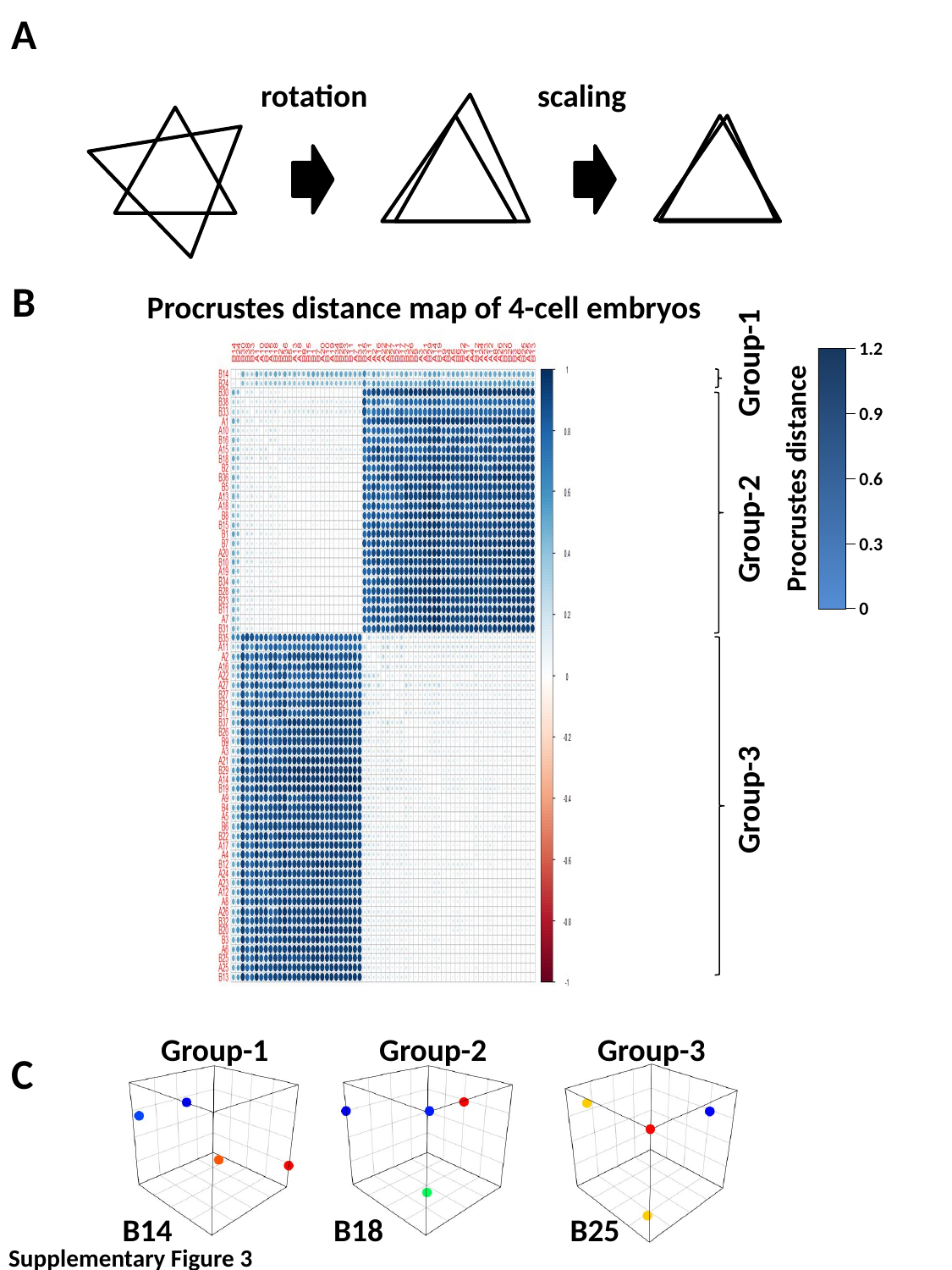

A
rotation
scaling
B
Procrustes distance map of 4-cell embryos
1.2
0.9
Procrustes distance
0.6
0.3
0
Group-1
Group-2
Group-3
Group-1
Group-2
Group-3
C
B14
B18
B25
Supplementary Figure 3

## Slide 4
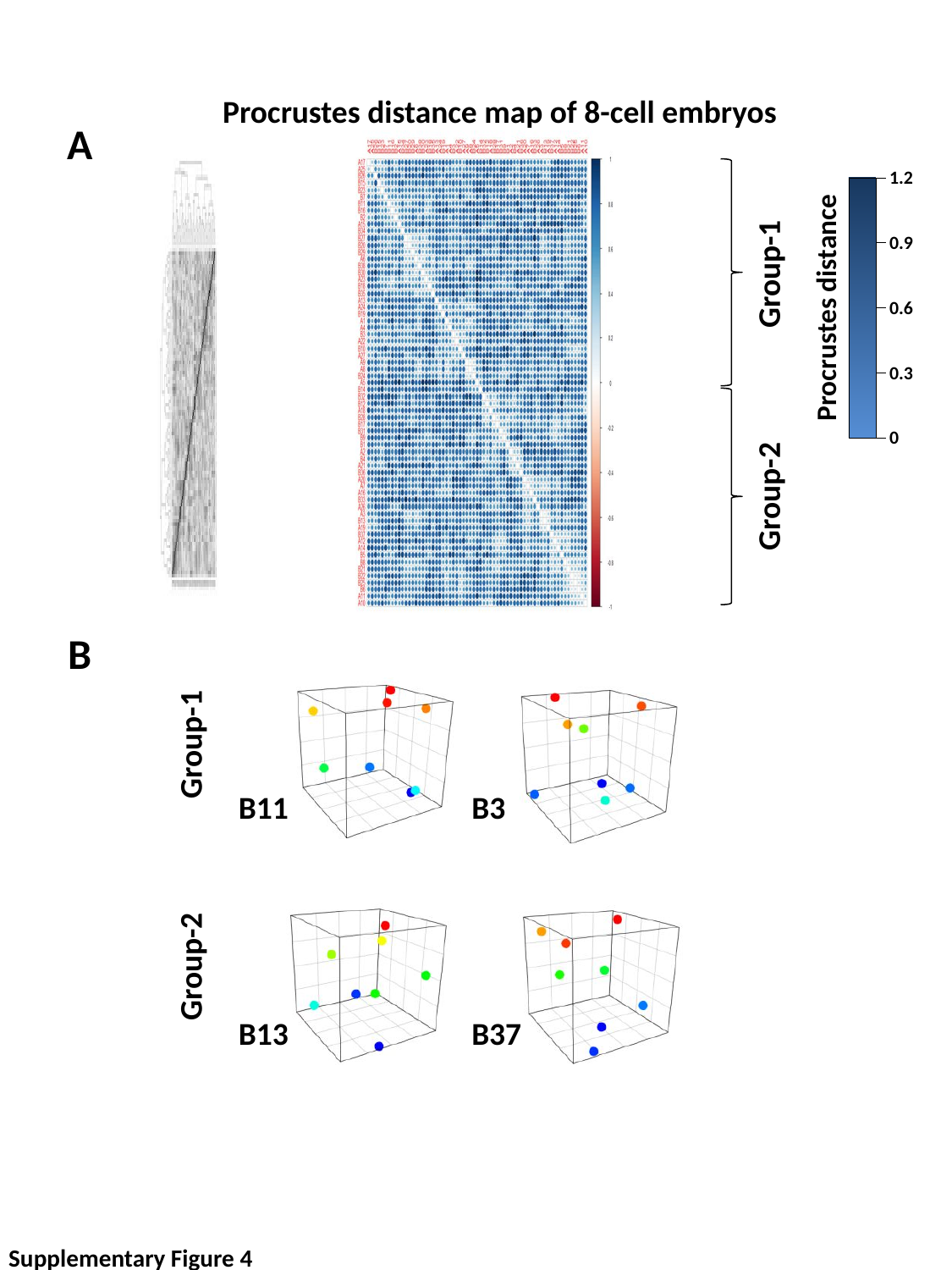

Procrustes distance map of 8-cell embryos
A
1.2
0.9
Procrustes distance
0.6
0.3
0
Group-1
Group-2
B
Group-1
B11
B3
Group-2
B13
B37
Supplementary Figure 4

## Slide 5
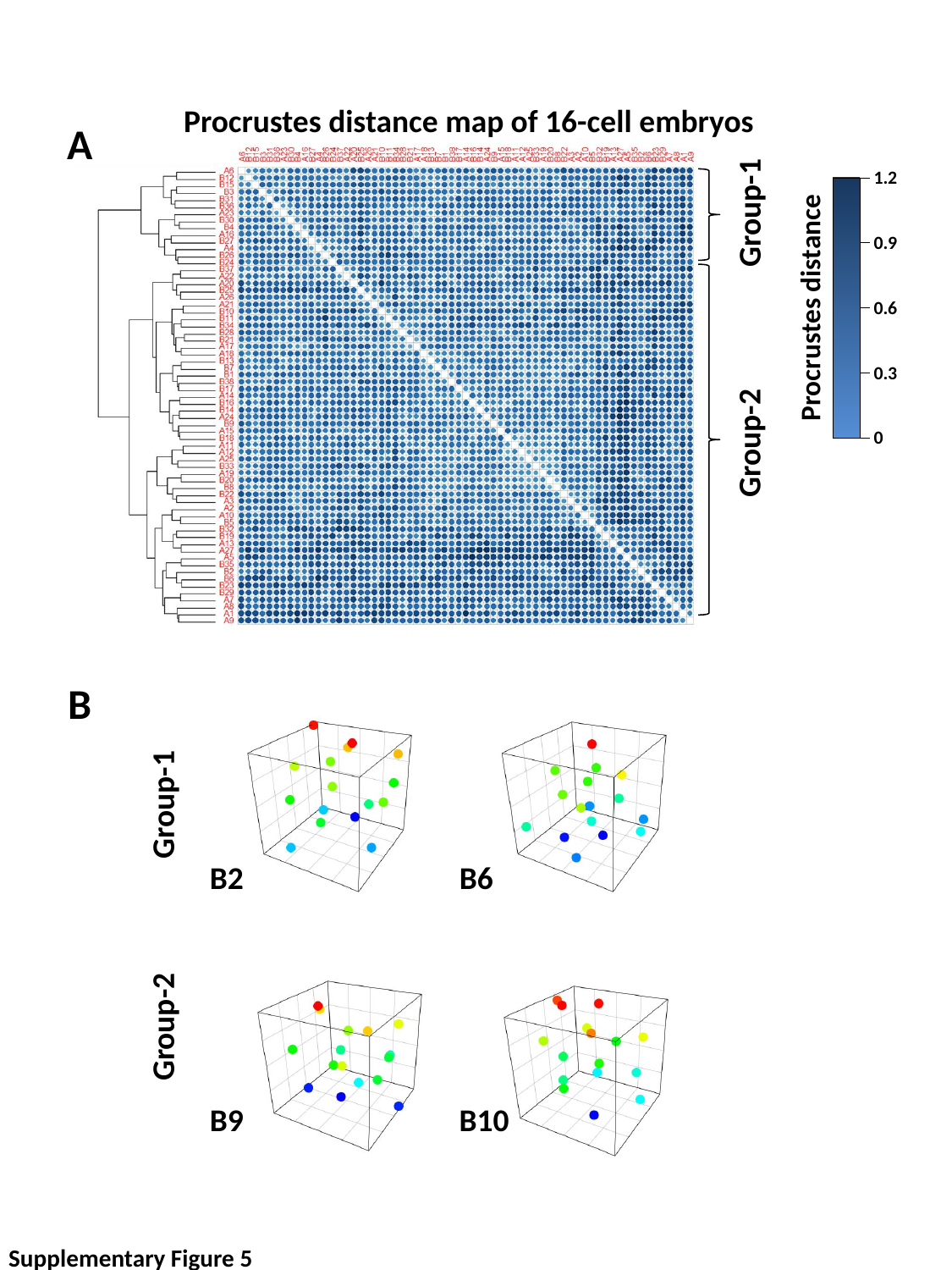

Procrustes distance map of 16-cell embryos
A
1.2
0.9
Procrustes distance
0.6
0.3
0
Group-1
Group-2
B
Group-1
B2
B6
Group-2
B9
B10
Supplementary Figure 5

## Slide 6
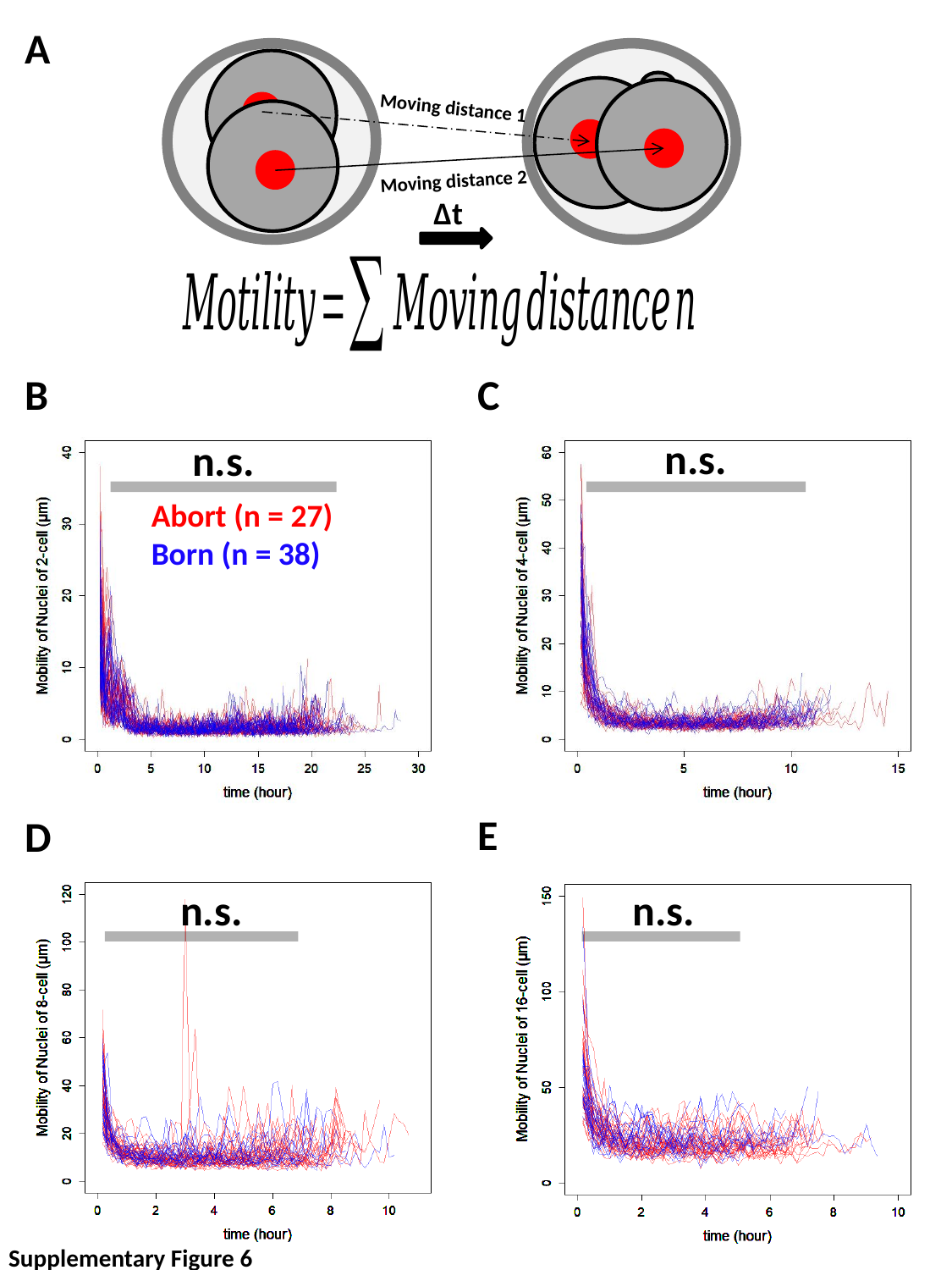

A
Moving distance 1
Moving distance 2
Δt
B
C
n.s.
n.s.
Abort (n = 27)
Born (n = 38)
E
D
n.s.
n.s.
Supplementary Figure 6

## Slide 7
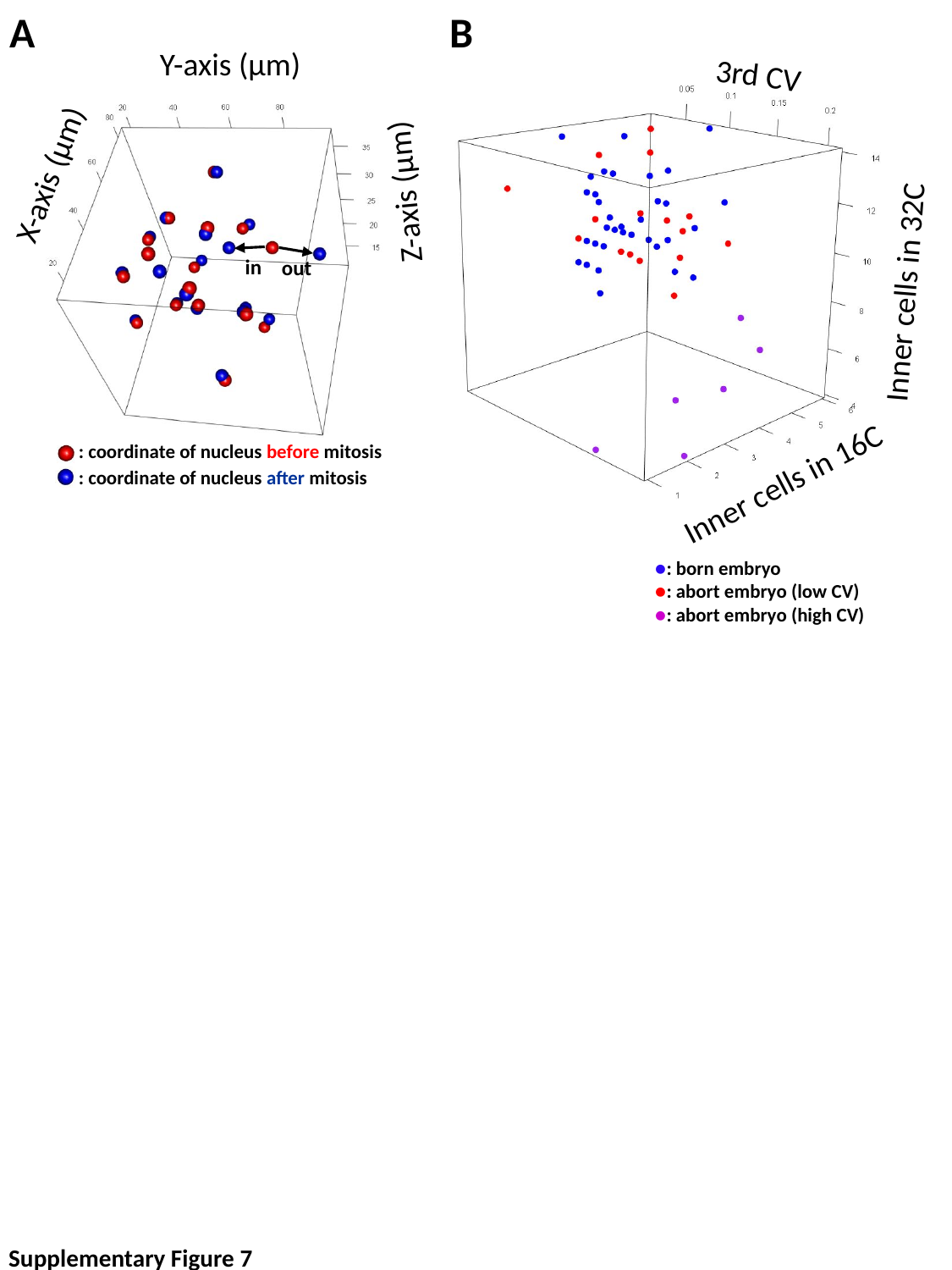

B
A
Y-axis (µm)
in
out
: coordinate of nucleus before mitosis
: coordinate of nucleus after mitosis
X-axis (µm)
Z-axis (µm)
3rd CV
Inner cells in 32C
Inner cells in 16C
●: born embryo
●: abort embryo (low CV)
●: abort embryo (high CV)
Supplementary Figure 7

## Slide 8
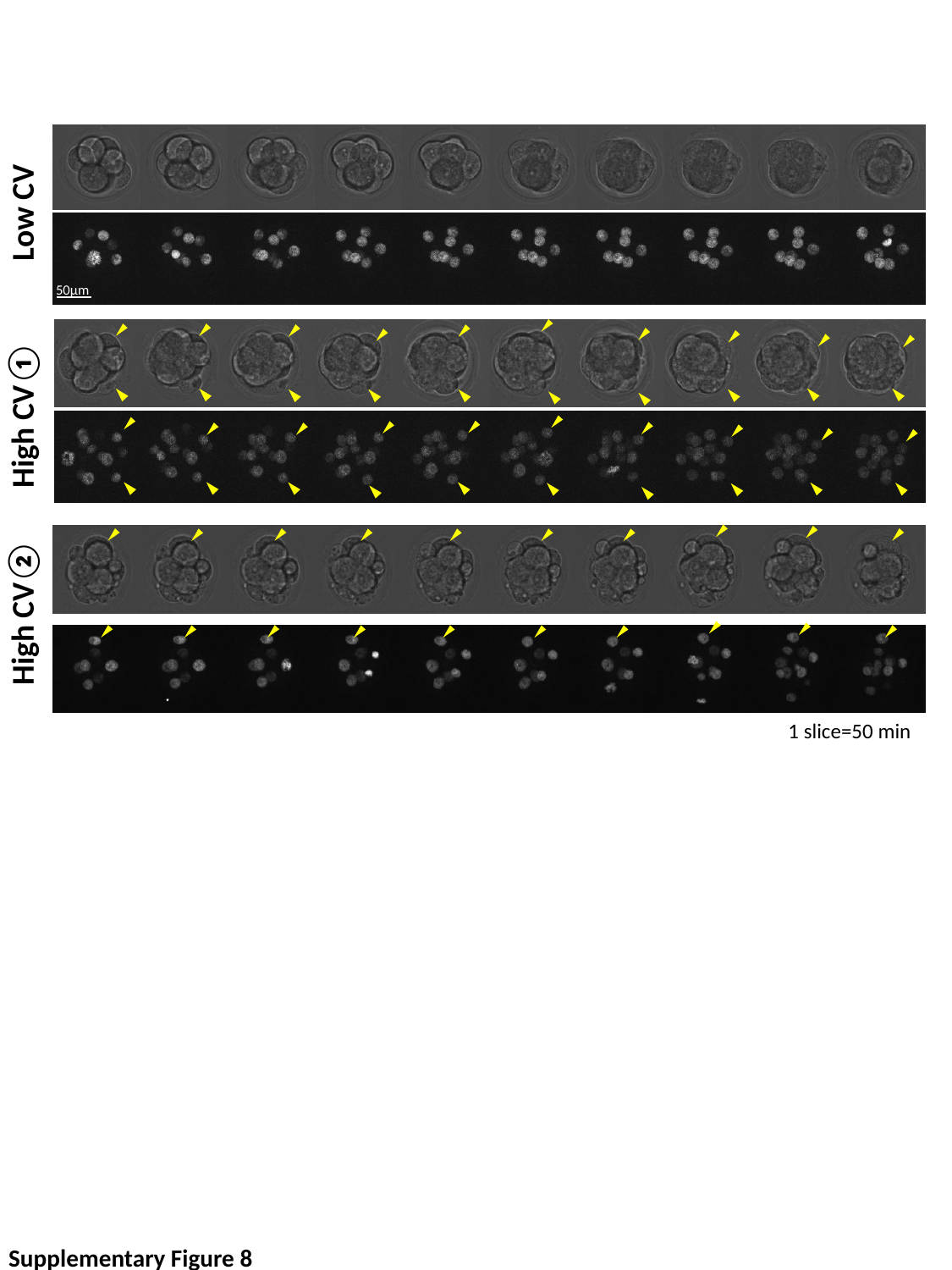

Low CV
50µm
High CV①
High CV②
1 slice=50 min
Supplementary Figure 8
