# Supplementary material for: Asynchronous division at 4–8-cell stage of preimplantation embryos affects live birth through ICM/TE differentiation
Source: Sci Rep. 2022 Jun 7;12:9411. doi: 10.1038/s41598-022-13646-8 (PMC9174281; doi:10.1038/s41598-022-13646-8)
Supplement: Supplementary file 5 — Supplementary Information 4. [file 41598_2022_13646_MOESM5_ESM.docx]

**Supplemental text**

By measuring the distance between the barycentric coordinates of the nucleus, which is one of the parameters that characterize the shape of the embryo, born/abort embryos were compared. First, the distance of the barycentric coordinates of the nuclei at the 2-cell stage were measured (Supplemental Figure 1A, 1B). Immediately after the division, the two nuclei were farthest apart. Subsequently, they closed some distance (Supplemental Figure 1C). The peak value, the largest distance value, was compared between born and abort cases, but there was no significant difference (Student’s t-test, P = 0.88, Supplemental Figure 1D).

The CV of the distance between the nuclei of each blastomere of 4, 8, 16, and 32-cell embryos was measured to express the shape as a single parameter. The CV of the nuclear distance in the 4, 8, 16, and 32-cell periods was compared in the born and abort cases. Although the CVs of the nuclear distances of 4,8, 16, and 32 cells in average time duration (4-cell: 11 h, 8-cell: 7-h, 16-cell: 5 h, 32-cell: 3 h) were compared in 10 min increments, the CV of all intervals showed no significant difference (Wilcoxon rank-sum test, P >0.1; Supplemental Figure 2–D; born:abort = 38:27).

To compare the arrangement of blastomeres, Procrustes analysis (39) of 4, 8, and 16-cell embryos was performed. The arrangement of the blastomeres just before the division was used for comparison. Two embryos were superimposed by moving, rotating, and scaling based on their size, and the difference in shape between embryos was evaluated by the Procrustes distance (Supplemental Figure 3A). When the distance between embryos was divided into clusters and a Procrustes distance map was created (Supplemental Figure 3B), three groups were clearly seen, i.e. group1: flat type, group2: non-regular tetrahedron type, group3: non-regular tetrahedron type (Supplemental Figure 3C). The ratio of born/abort in each group was: group1 (B2:A0), group2 (B18:A8), group3 (B18:A19) There was no significant difference among the groups (prop-test, P = 0.1268). The same analysis was performed on 8- and 16- cells, but a clear group did not appear as with 4-cell groups (Supplemental Figures 4 and 5). Therefore, we adopted a rough method where all embryos were divided into two groups by clustering and were compared to the ratio of born and abort outcomes. As a result, there was no bias in the ratio of born and abort in both 8- and 16- cells (prop-test; 8-cell: P = 0.7535, 16-cell: P = 0.6751).

The movement of the barycentric coordinates of the nucleus was measured. The sum of the movement distance of nuclei was measured every 10 min and compared in “born case” and “abort case” (Supplemental Figure 6A). On comparing the movement distances during 2, 4, 8, 16-cell time average duration (21h, 11h, 7h, 5h), no significant difference was found in all the intervals (Wilcoxon rank-sum test, P> 0.1; Supplemental Figure 6B–E).

**Supplemental Figure legends**

**Supplemental Figure 1 There was no significant difference in the distance between nuclei at the two-cell stage between born and aborted embryos**

**(A)** Distance between nuclei of individual 2-cell stage embryos. The time immediately after the first division, is set to t = 0. Red and blue lines represent abort and born embryos, respectively.

**(B)** Average distance between nuclei of 2-cell stage embryos. Pink and sky-blue lines represent abort and born embryos, respectively. The arrow indicates the peak value.

**(C)** Nuclear position at peak. The blue arrow represents the peak.

**(D)** Comparison of peak value of nuclei of the 2-cell stage between born and abort embryos.

**Supplemental Figure 2 The coefficient of variation of internuclear distance of 4, 8, 16 cell embryos is not significantly different between born and aborted embryos**

**(A)** Comparison of the CV of internuclear distance at the 4-cell stage. Embryos showing CV >0.2 are close to the shape of a non-tetrahedron or planer, and embryos showing CV <0.1 are close to the shape of a tetrahedron. The red and blue lines indicate abort and born, respectively.

**(B)** Comparison of the CV of internuclear distance at the 8-cell stage. CV = 0.21 represents a cube shape.

**(C)** Comparison of CVs of 16-cell stage internuclear distance.

**(D)** Comparison of the CV of internuclear distance at the 32-cell stage.

**Supplemental Figure 3 Procrustes analysis of 4-cell embryos**

**(A)** Schematic diagram of Procrustes analysis.

**(B)** Procrustes distance map of 4-cell embryos.

**(C)** Groups of 4-cell stage shapes.

**Supplemental Figure 4 Procrustes analysis of 8-cell embryos**

**(A)** Procrustes distance map of 8-cell embryos.

**(B)** Groups of 8-cell stage shapes.

**Supplemental Figure 5 Procrustes analysis of 16-cell embryos**

**(A)** Procrustes distance map of 16-cell embryos.

**(B)** Groups of 16-cell stage shapes.

**Supplemental Figure 6 Motility of blastomeres of 2, 4, 8, 16-cell embryos was not significantly different between born and abort**

**(A)** Schematic diagram of motility definition and calculation.

**(B)** Comparison of 2-cell motility between born and abort.

**(C)** Comparison of 4-cell motility between born and abort.

**(D)** Comparison of 8-cell motility between born and abort.

**(E)** Comparison of 16-cell motility between born and abort.

**Supplemental Figure 7** **Measurement of inner/outer cell numbers by observing the direction of division**

**(A)** Visualization of barycentric coordinates of all nuclei in a time frame when the number of cells increases.

**(B)** Three-dimensional plot of third CV, inner cells in the 16-cell stage (16C), inner cells in the 32-cell stage (32C). Blue dots are born embryos. Red dots are aborted embryos with low CV. Purple dots are aborted embryos with high CV.

**Supplemental Figure 8 The absence of flattening in not-all cells was not caused by cell division**

From left to right, live-cell images taken every 50 minutes are shown. The yellow arrowheads on the high-CV embryo indicate the blastomeres that were not flat. These cells were not observed to be in the M-phase.

**Supplemental Movie 1 A typical movie of an embryo with delayed compaction**

The compaction of some blastomeres is delayed.

**Supplemental Table 1 Extracted coordinates of born mouse embryos**

X, Y, and Z indicate the XYZ coordinates. Frame indicates the time frame (frame/10 min). Each row indicates a single nucleus. The names of the sheets are defined by the following rule: [born or abort] [the number of experiments] − [the number of drops of medium] − [the number of embryos].

**Supplemental Table ２ Extracted coordinates of abort mouse embryos**

X, Y, and Z indicate the XYZ coordinates. Frame indicates the time frame (frame/10 min). Each row indicates a single nucleus. The names of the sheets are defined by the following rule: [born or abort] [the number of experiments] − [the number of drops of medium] − [the number of embryos].
